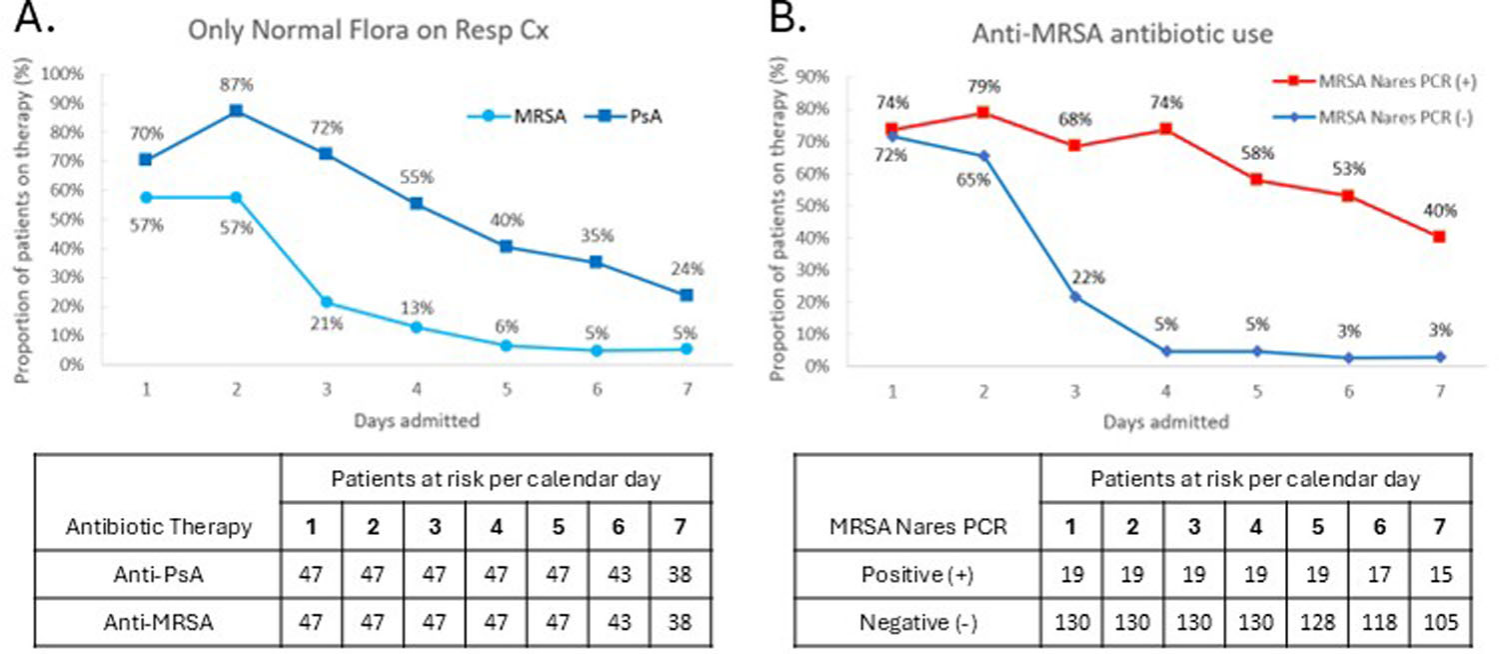# 119 Changes to Trends in Reported Long-Term Care Norovirus Outbreaks in the United States during 2015-2025

**DOI:** 10.1017/ash.2026.10533

**Published:** 2026-06-23

**Authors:** David Dickson, Marisa Holubar, David Ha

**Affiliations:** 1 Stanford University; 2 Stanford University School of Medicine; 3 Stanford Medicine

## Abstract

**Background:** Community acquired pneumonia (CAP) is a common and frequently serious infection. Guidelines recommend empiric anti-MRSA and/or anti-pseudomonal therapy in patients with severe CAP who have previously grown those organisms in the last year or recently received intravenous antibiotics, as well as respiratory cultures for those on anti-MRSA and/or anti-pseudomonal antibiotics or with severe CAP. We previously showed overutilization of anti-MRSA and anti-pseudomonal antibiotics and underutilization of respiratory cultures in patients with CAP admitted to the ICU. Anti-MRSA antibiotics were readily de-escalated with negative MRSA nares PCR testing, but anti-pseudomonal therapy persisted even with respiratory culture data demonstrating absence of Pseudomonas. These findings were disseminated to ICU teams via departmental talks, bi-weekly in-person stewardship handshake, and an EMR nudge (for anti-pseudomonal prescribing). Here, we performed a timed reassessment of antibiotic use in this population following these educational initiatives while investigating additional factors that may influence empiric choice and de-escalation practices. Methods We performed an IRB approved single-center retrospective chart review including adult patients who were admitted to the ICU and received intravenous pneumonia-directed antibiotics within two calendar days of admission between January 1st, 2024 and August 31st, 2025. Patients were excluded if they were transferred from an acute care facility, were discharged or transitioned to comfort care within 48 hours of admission, had lung transplant or BMT, CF, PJP, lung abscess, empyema, suspicion for extra-pulmonary infection, any recent neutropenia (ANC<500), or were chronically ventilated. Data was collected from internal dashboards and manual electronic medical record review. Results Of the 658 encounters reviewed, 234 patient encounters were included in our primary analysis. 80% (187/234) and 77% (179/234) of patients received empiric anti-pseudomonal and anti-MRSA therapy, while only 28% (52/187) and 30% (54/179) had risk factors for Pseudomonas and MRSA, respectively. 43% (89/208) of patients with an indication for respiratory culture collection had one obtained within 48 hours of admission. Of the patients who grew only normal flora (with reported absence of MRSA and/or Pseudomonas) from their respiratory cultures, de-escalation of anti-pseudomonal antibiotics lagged behind that of anti-MRSA antibiotics (Figure 1A). Anti-MRSA de-escalation was associated with negative MRSA Nares PCR testing (Figure 1B). Conclusion Educational interventions were ineffective strategies to improve guideline concordant management of critically ill patients with CAP. Respiratory cultures remain infrequently utilized. Growth of normal flora on respiratory culture rarely results in de-escalation away from anti-pseudomonal therapy the way that negative MRSA nares testing does for anti-MRSA therapy.

**Figure:**